# Experimental Models to Study Podocyte Biology: Stock-Taking the Toolbox of Glomerular Research

**DOI:** 10.3389/fped.2018.00193

**Published:** 2018-07-13

**Authors:** Henning Hagmann, Paul T. Brinkkoetter

**Affiliations:** Department II of Internal Medicine, Center for Molecular Medicine Cologne, University of Cologne, Cologne, Germany

**Keywords:** model organism, kidney diseases, podocyte, mechanosensation, glomerular filtration barrier

## Abstract

Diseases affecting the glomeruli of the kidney, the renal filtration units, are a leading cause of chronic kidney disease and end-stage renal failure. Despite recent advances in the understanding of glomerular biology, treatment of these disorders has remained extraordinarily challenging in many cases. The use of experimental models has proven invaluable to study renal, and in particular, glomerular biology and disease. Over the past 15 years, studies identified different and very distinct pathogenic mechanisms that result in damage, loss of glomerular visceral epithelial cells (podocytes) and progressive renal disease. However, animal studies and, in particular, mouse studies are often protracted and cumbersome due to the long reproductive cycle and high keeping costs. Transgenic and heterologous expression models have been speeded-up by novel gene editing techniques, yet they still take months. In addition, given the complex cellular biology of the filtration barrier, certain questions may not be directly addressed using mouse models due to the limited accessibility of podocytes for analysis and imaging. In this review, we will describe alternative models to study podocyte biology experimentally. We specifically discuss current podocyte cell culture models, their role in experimental strategies to analyze pathophysiologic mechanisms as well as limitations with regard to transferability of results. We introduce current models in *Caenorhabditis elegans, Drosophila melanogaster*, and *Danio rerio* that allow for analysis of protein interactions, and principle signaling pathways in functional biological structures, and enable high-throughput transgenic expression or compound screens in multicellular organisms.

## Introduction

**Figure 1:**
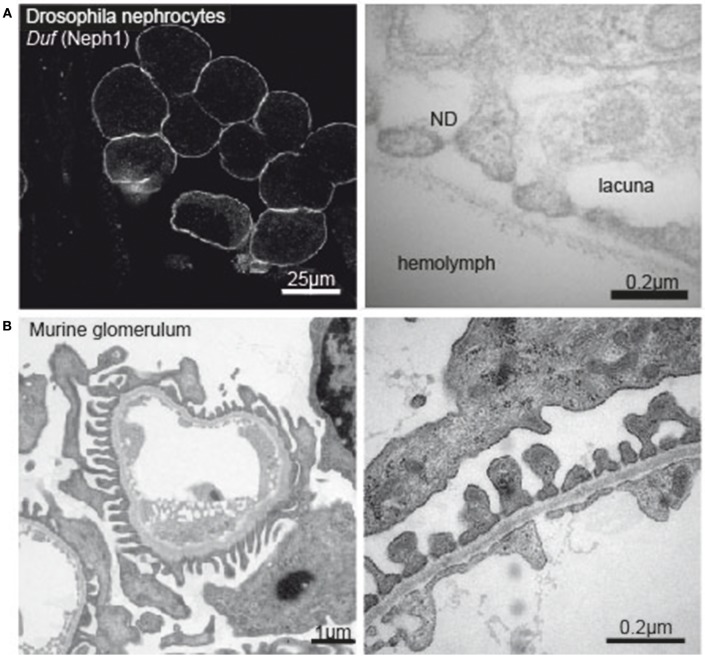
**(A)** Immunofluorescence image of *Drosophila* nephrocytes stained with *Duf* (Neph1)-specific antibody (left tile) and electronmicrograph of the nephrocyte apical membrane showing lacunae, slit diaphragm and basement membrane (right tile). **(B)** Electron micrograph of a murine glomerular capillary (left tile) and higher magnification of the filtration barrier consisting of glomerular endothelium, basement membrane and podocyte foot processes (right tile).

Chronic kidney disease (CKD) is becoming an increasingly prevalent condition affecting almost 10% of the population in Western societies. The majority of kidney diseases that progress to end stage renal failure start in the glomerulus, the renal filtration unit, as a consequence of a very limited capacity of glomeruli for regeneration and the limited ability of terminally differentiated glomerular podocytes for self-renewal (1). The glomerular filtration barrier consists of three anatomic layers: fenestrated endothelial cells, the glomerular basement membrane and podocytes, post-mitotic epithelial cells located at the outer aspect of the capillary loops (Figure 1B). These cells enwrap the glomerular capillaries with their primary and secondary processes and form the outer layer of the filtration apparatus. All three layers contribute substantially to the glomerular filtration barrier and can be affected in human disease. Due to their exposed anatomic localization podocytes are constantly challenged not only by oxygen radicals, cytokines, immune complexes and inflammatory processes but also by mechanical forces. Podocyte damage plays a pivotal role in most, if not all, glomerular diseases that result in glomerulosclerosis (2). As podocyte loss cannot easily be compensated by cell proliferation the cells undergo hypertrophy, autophagy, and/or dedifferentiation depending on the injurious insult (3). Podocyte hypertrophy and the increase in cellular size and the covered area of the GBM represent protective measures to ascertain proper glomerular function. In contrast, dedifferentiation is considered to be maladaptive resulting in albuminuria and persistent podocyte loss. It has to be stressed, that the onset of albuminuria and even nephrotic range proteinuria does not require podocyte depletion. Mere cytoskeletal rearrangements, i.e., foot process effacement, are sufficient to cause massive albuminuria as often seen in patients with minimal change disease (MCD). A condition which lacks evidence of pathology in light microscopy but presents with vast foot-process effacement in electron microscopy. In the event of progressive or severe glomerular disease podocyte loss is the clue and patients develop massive albuminuria in combination with irreversible scarring, i.e., glomerulosclerosis (1, 4–7). Importantly, albuminuria and chronic kidney disease are independently associated with an increased risk for end stage renal failure and cardiovascular disease (8–10).

The finding that independent pathways and pathogenic principles contribute to the identical glomerular phenotype described as focal segmental glomerulosclerosis (FSGS) seems trivial but is of major importance. Podocytes are firmly attached to the underlying glomerular basement membrane and form a unique cell-cell contact to foot processes of neighboring podocytes, a cell junction called slit diaphragm. This specialized cell-cell contact is not only an integral part of the glomerular filtration barrier but also serves as signaling hub to regulate podocyte function (11). Over the past several years, various constituents of the podocyte slit diaphragm cell junction have been identified leading to the concept that the proteins at the slit diaphragm regulate podocyte biology through active signaling. The slit diaphragm bridges the distance between two adjacent foot processes, thus allowing formation of a filtration slit. In severe podocyte damage, the slit diaphragm disappears and podocytes simplify structure and shape due to cytoskeletal alterations, a process called foot process effacement (8, 10). Until recently, the function of the glomerular filtration barrier and the pathogenesis of proteinuria have not been well understood. This has changed with the identification of gene defects in (rare) human genetic diseases known to cause congenital or childhood steroid-resistant nephrotic syndrome and progressive glomerulosclerosis [for review see 9]. These studies identified distinct deregulated pathways that independently contribute to podocyte injury and, potentially, loss of podocytes. Podocyte depletion has long been known to be the culprit of glomerulosclerosis and progressive loss of renal function (12–14). As multiple different pathogenic mechanisms result in proteinuria and FSGS lesions in kidney biopsies, it is not surprising that several clinical trials including all FSGS patients failed to provide new MCD/FSGS treatment options, e.g. the NIH has spent multimillion dollars on clinical trials that did not yield a single new drug for MCD/FSGS patients (15).

The advent of modern genetics with the development of animal models with cell specific gene manipulation including gene deletions and transgenic gene expression together with systems biology has deepened our understanding of the biology and physiology of the renal filtration barrier in states of health and disease. Despite our tremendous advances in understanding glomerular function and the contribution of the specific anatomic compartments to the renal filtration barrier, essential questions remain to be addressed as treatment of glomerular disorders is still unspecific and primarily based on various immunosuppressive regimens, including glucocorticoids or blockade of the renin-angiotensin-aldosterone system (15).

In the past, the use of experimental models has proven invaluable to study renal, and in particular, glomerular biology and disease. Even after the introduction of novel gene editing techniques, mouse models are time consuming. The relatively long reproductive cycle, high keeping cost, and not least the regulatory standards make these models less flexible. In this review, we will describe alternative models to study podocyte biology experimentally.

## Podocyte cell culture

Podocyte cell culture models were the first models to study podocyte biology and are still widely used (16, 17) as gene and protein expression as well as environmental cues can be easily manipulated *in vitro* for mechanistic analyses (18, 19). Multiple human, mouse, and rat podocyte cell lines have been generated in the past (16, 17, 20–22). Most groups rely on immortalized mouse or human podocyte cell lines which are cultured under proliferative (33°C) and growth-restrictive (37°C) conditions. These studies promoted our understanding of glomerular diseases (19) as well as the cytoskeletal regulation (23), cell cycle control (24), cell death mechanisms (25), signaling pathways (26, 27) as well as protein degradation (28).

However, certain limitations apply and have to be taken into account when translating experimental findings from cultured podocyte cell lines; podocytes in culture are cultivated on petri-dishes as a monolayer in the absence of mesangial and endothelial cells in close proximity. Podocytes in culture do not encounter mechanical stretch nor the flow of primary urine filtrate (29). Hence, it is not surprising that podocyte cell lines do not form secondary processes with slit diaphragms in-between neighboring cells and show only a very limited expression of specific marker proteins including nephrin (30), podocin (31), or transient receptor potential cation channel 6 (26, 32, 33).

In an attempt to further characterize these widely used work horses of podocyte research our group recently applied modern MS/MS technologies and created a comprehensive map at a depth of more than 7,000 proteins expressed in proliferating and differentiated cultured podocytes *in vitro* (26). To this end, we examined an immortalized mouse podocyte cell line kindly provided by S. Shankland (Seattle, WA) as well as a conditionally immortalized human podocyte cell line obtained from M. Saleem (Bristol, UK), both are widely used cell-culture models generated by either isolation of primary podocytes from the immortomouse (34) or by retroviral transfection of primary human podocytes with a temperature-sensitive SV40 large T-cell antigen (35) (Table 1). The temperature shift from 33 to 37°C induces in both cell lines a proteostatic shift. Undifferentiated podocytes express high abundance of proteasomal proteins while differentiated podocytes express high abundance of lysosomal proteins. Additional studies using pulsed stable isotope labeling by amino acids in cell culture (pSILAC) and protein degradation assays determined protein dynamics and half-lives and revealed a globally increased stability of proteins in differentiated podocytes. Mitochondrial, cytoskeletal and membrane proteins were particularly stabilized in differentiated podocytes. However, the expression levels of so-called podocyte marker genes or podocytopathy gene products varied significantly as compared to primary cells. Highest levels were detected for Actin-regulating proteins comprising Myosin-9 (MYH9) (36), rho GDP-dissociation inhibitor 1 (ARHGDIA) (37) and alpha-actinin-4 (ACTN4) (5). Out of 15 podocytopathy-associated genes linked to cytoskeletal function 8 were expressed in the cultured cells (MYH9, ARHGDIA, ACTN4, anillin (ANLN), inverted formin-2 (INF2), unconventional myosin IE (MYO1E), synaptopodin (SYNPO), and podocalyxin (PODXL) (22, 38–41). With respect to basement membrane proteins 4 out of 8 were quantified in undifferentiated and differentiated human podocytes [CD151 antigen (CD151), integrin alpha-3 (ITGA3), integrin beta-4 (ITGB4), and laminin subunit beta-2 (LAMB2) (20, 42, 43). In contrast, only one out of six slit diaphragm proteins (CD2-associated protein (CD2AP) (44) could be detected.

**Table 1:** Expression profiles of podocyte-specific marker proteins known as determinants of cell specificity as suggested by Shankland et al. (26).

**Protein name**	**Gene name**	**Cultured Human Podocyte Proteome** (45)		**Cultured Mouse Podocyte Proteome** (26)	
		**33°C**	**37°C**	**33°C**	**37°C**
**CYTOSKELETAL PROTEINS**					
Myosin-2	MYH2; MYHSA2	–	–	–	–
Podocalyxin	POD+L	+	+	+	+
Synaptopodin	SYNPO	+	+	+	+
Alpha-actinin-4	ACTN4	+	+	+	+
**BASEMENT MEMBRANE PROTEINS**					
Integrin alpha-3	ITGA3	+	+	+	+
Integrin beta-1	ITGB1	+	+	+	+
Integrin-linked protein kinase	HEL-S-28; ILK	+	+	+	+
**SLIT DIAPHRAGM PROTEINS**					
CD2-associated protein	CD2AP	+	+	+	+
Nephrin	NPHS1	–	–	–	–
Cytoplasmic protein NCK1	NCK1	+	+	+	+
Cytoplasmic protein NCK2	NCK2	+	+	+	+
Podocin	NPHS2	–	–	–	–
Short transient receptor potential cation channel 6	TRPC6	–	–	–	–
**MEMBRANE PROTEINS**					
Type-2 angiotensin II receptor	AGTR2	–	–	–	–
T-lymphocyte activation antigen CD80 (Activation B7-1 antigen)	CD80	–	–	–	–
P-Cadherin	CDH3	–	–	–	–
**OTHER**					
Transcription factor 21 (podocyte-expressed 1)	TCF21 (POD1)	–	–	–	–
Transforming growth factor beta-1/2	TGFB1/2	+	+	+	+
Vascular endothelial growth factor A	VEGFA	–	–	–	–
Wilms tumor protein 1	WT1	–	–	–	–

*Respective proteins are either marked as expressed (“+”) or not expressed (“–”) in the two examined cell culture conditions (33°C = undifferentiated, and 37°C = differentiated cells). According to Schroeter et al. (45)*.

## Model organisms to study podocyte biology

*Caenorhabditis elegans* with its short life cycle, completely established cell lineage including a neuronal map (connectome), compact and fully mapped genome, uncomplicated genetic modification by feeding of RNAi-expressing *E. coli*, and cost-effective keeping is a prime model for cell biology (46). In addition and in contrast to other multicellular model organisms, (genetically modified) individuals can be easily frozen, stored for the longer term, and revived with immediate capability to reproduce.

These characteristics make the nematode an ideal model to study signaling pathways and functionality of proteins in a multicellular organism and represent an advantage as compared to mammalian models.

Principle signaling pathways like e.g., the insulin/mTOR signaling cascade are generally well-conserved across species (47). Along this line, the observation that the insulin/mTOR pathway is induced and refers damage in podocytes with mitochondrial dysfunction due to the loss of Prohibitin-2 (PHB-2) was substantiated in *C. elegans* using worm strains expressing fluorescence labeled DAF-16 (48). After heat-shock DAF-16 is pooled in the nuclear compartment. Redistribution of DAF-16 to the cytoplasm depends on the activity of the insulin pathway. Consistently with data from podocyte-specific PHB-2 knockout mice, *phb-2*-deficient worms showed accelerated recovery of the DAF-2 (insulin receptor) mediated cytosolic redistribution of DAF-16.

However—with regard to glomerular research—*C. elegans* does not contain a filtering excretory organ homolog to the mammalian glomerulum. Nevertheless, ortholog genes in analogs structures can be studied in the nematode to understand principle mechanisms of podocyte morphogenesis and podocyte slit-diaphragm function.

The mammalian slit diaphragm is composed of the transmembranous immunoglobulin family proteins nephrin and Neph1. Nephrin and Neph1 are lipid raft associated proteins that refer outside-in signals by tyrosin phosphorylation. Mutations in the nephrin encoding gene NPHS1 or lack of NEPH1 lead to defective assembly of the foot processes and loss of the slit diaphragm which becomes evident as (congenital) nephrotic syndrome (49, 50). The adhesion molecules nephrin and Neph1 are well conserved across species. In *C. elegans* orthologs of Neph1 and Nephrin are SYG-1 and SYG-2, respectively. SYG-1 and SYG-2 refer cell-cell recognition in synapse development between the hermaphrodite specific neuron (HSN) and specialized epithelial guidepost cells adjacent to the nematode's vulva muscle cells medially in the hermaphrodites' soma. The two HSN (HSNL and HSNR) localize to the lateral aspects in the middle of the nematode and protrude their axonal processes ventrally, where they innervate the vulvar muscle cells and provide the neuronal circuit required for egg laying. Interaction of SYG-1 on the HSN axon with SYG-2 expressed on guidepost cells initiates intracellular signaling processes in HSN to trigger synapse formation and maintenance (51, 52). Mutations in either *syg-1* or *syg-2* fail to exhibit functional synapses due to aberrant placement of presynaptic sites (53, 54). Interestingly, heterologous expression of mammalian nephrin or Neph 1, −2 or −3 can rescue phenotypes of mutant *syg-1* or *syg-2* (55, 56). The cytoplasmic tail of SYG-2 is required for subcellular trafficking of SYG-2 itself, whereas the cytoplasmic domain of SYG-1 is required for synapse formation but dispensable in later stages (57). Although elegant ultrastructural analyses in mammalian and avian glomeruli have challenged the concept of heterophilic nephrin and Neph1 interaction at the slit diaphragm, synapse formation at the HSN in *C. elegans* may represent a suitable model to study signaling mechanisms at the cytoplasmic domains of nephrin and Neph1 by visualization of synaptic vesicles in SNB-1::YFP transgenic worms.

Another example for the utility of *C. elegans* in glomerular research is based on the homology of mammalian podocin and *C. elegans* MEC-2. Podocin is an essential constituent of the mammalian slit diaphragm complex, whereas MEC-2 is part of the mechanosensory complex of *C. elegans* sensing gentle touch. Both stomatin-like proteins share a central stretch of hydrophobic amino acids which refers membrane association while the amino and the carboxy terminal ends face the cytoplasm. The highly conserved PHB domain mediates homophilic interactions and lipid binding via palmitoylation, creating the microenvironment that regulates signaling via the associated ion channel proteins TRPC6 in mammals and the DEG/ENaC channel MEC-4/MEC-10 in *C. elegans* (58–60). This regulatory role of MEC-2 can not only be assessed by quantification of mechanoreceptor channel currents but also in functional *in vivo* assays measuring sensitivity to gentle touch in adult hermaphrodites (61). In addition, regular localization of MEC-2 and other components of the mechanosensory complex in a characteristic punctate pattern on the six mechanosensory neurons of *C. elegans* can be evaluated by staining with MEC-2 specific antibodies or employing MEC-4::YFP transgenic worms (28, 62). Identifying co-localization of the primarily mitochondrial protein Prohibitin 2 (PHB-2) and MEC-4 in mechanosensory punctae of touch receptor neurons in *C. elegans* as well as partial loss of touch sensitivity in PHB-2 knock down worms helped to establish the role of PHB-2 as a slit-diaphragm protein (63). In a recent paper, the ubiquitin ligase Ubr4 has been shown to control podocin protein stability and conservation of this molecular mechanism could be confirmed for MEC-2 in *C. elegans* assays, where the loss of the Ubr4 ortholog C44E4.1 (ubr-4) resulted in a more dispersed staining pattern of MEC-2 positive punctae (28).

With regard to glomerular research, studying *C. elegans* is instrumental as a functional read out for protein interactions, trafficking and protein turnover as well as signaling of conserved pathways in a multicellular organism. This holds true especially for mammalian podocin, nephrin, Neph 1, and their orthologs. A weakness of the nematode as a model of conserved principles in mammalian (patho-)physiology as well as for compound screens is the lack of organs homolog to heart, liver, central nervous systems, and of course filtering organs like the kidney.

*Drosophila melanogaster* evolved as another high-capacity model organism for glomerular research. Cell type specific gene inactivation and editing make the fly a versatile, adaptable, and expedite model. The reproductive cycle is around 12 days.

Similar to the nematode the Drosophila model allows to study protein interactions in functional biologic structures. In the fly adhesion molecules ortholog to mammalian nephrin and Neph proteins called sticks'n stones/hibris (sns/hbs) and kirre/dumbfounded (duf) are involved in cell-cell recognition, and cellular signaling events to control adhesion, cell shaping, and programmed cell death during eye development in the fly. During embryonal development Drosophila's facet eyes develop from a single layered epithelium and remain undifferentiated during almost the entirety of larval stages. In the final differentiation steps heterophilic interactions of sns/hbs and duf determine cell fate specification and are required for patterning and separation of ommatidea (64, 65).

During the last decade, research explored *Drosophila* nephrocytes as a novel tool of cell biology. The term was initially coined by Bruntz and Kowalsky, who discovered ammonia carmin absorbing cells around the heart, the digestive organs, and the nervous system in arthropods (66, 67). Nephrocytes are specialized filtrating cells with high endocytic activity, that may have detoxifying and sequestration function. In the adult fly there are two populations of nephrocytes. The Garland nephrocytes, which are assembled along the esophagus, and the pericardial nephrocytes, which palisade the heart tube. Nephrocytes are large cells with a surface laced by invaginations, so called labyrinthine channels, and covered by a continuous basement membrane. Near the apical surface the invaginations are abridged by a slit diaphragm (Figure 1A). Loss of the nephrin ortholog *sns* or the Neph ortholog *duf* results in loss of slit diaphragm structures, smaller lacunae and thickening of the basement membrane (68). Filtration across the pericardial nephrocyte slit diaphragm is limited to substances smaller than 70 kDa (69). Molecules that get filtered into the labyrinthine channels are taken up by the nephrocyte via endocytosis (70). In this regard the nephrocyte differs significantly from mammalian podocytes as to our current understanding. Filtration across the nephrocyte slit diaphragm as well as endocytosis can be assessed in assays on explanted nephrocytes employing different tracers, like e.g., GFP-, labeled albumin, horseradish peroxidase or dextrans of variable sizes (68, 69, 71, 72).

A novel transgenic fly model expressing secreted atrial natriuretic factor labeled with red fluorescent protein (ANF-RFP) from muscle cells and nephrocyte-specific green fluorescent protein (GFP) combined with the option of nephrocyte specific gene manipulation via a nephyrocyte specific Dot-Gal4 driver has implemented an expedite and reliable screening tool for genes associated with human disease (69). This model was employed to screen Drosophila orthologs of human monogenic nephrotic disease. Reassuringly, most of the pathogenic alterations were conserved in the fly (71, 73). Loss of Coq2 or Rab GTPases e.g., leads to loss of labyrinthine channels and loss of function in nephrocytes (71, 74).

In addition, rescue and overexpression studies with human orthologs are possible in Drosophila knockout models. Both eye and nephrocyte development were evaluated to identify a conserved amino acid motif in mammalian Neph1 to functionally replace Drosophila *duf*, whereas neither Neph2 nor Neph3 showed similar effects (75).

In general, the fly is an ideal model organism for high-throughput transgenic screens and rescue experiments, when variants of ortholog human genes are expressed in the respective drosophila knock out. The *in vivo* filtration assay or eye pattern formation serve as reliable and expedite readouts.

*Danio rerio*, the zebrafish, provides a third model organism of glomerular filtration. In striking contrast to the non-vertebrate *Drosophila* and *C. elegans models*, the zebrafish forms vascularized glomeruli in the pronephrons and the mesonephros during development with the mesonephros maintained in adult life. At the pronephros-stage the zebrafish kidney consists of two fused glomeruli with connection to the aorta draining primary urine into the pronephric tubuli followed by the pronephric ducts (76). The pronephros glomeruli already contain fenestrated endothelium, mesangial cells and podocytes that form a functional slit diaphragm (77). In addition, genome conservation of 70% between human and *Danio rerio* as well as versatile morphlino techniques, CRSPR/Cas-based gene editing and cross-species rescue models make the fish a primary model to study glomerular disease. Loss of zebrafish nephrin and podocin, which are specifically expressed in pronephric podocytes, leads to the loss of slit-diaphragms early in development (78). As in human nephrotic syndrome, pericardial edema, periocular edema, and general edema develops. Likewise disruption of zNeph1 or zNeph2 showed similar phenotypes (55). Besides structural analyses functional assays of glomerular filtration have emerged. Early on, the integrity of the filtration barrier was monitored qualitatively on fixed tissue after injections of large molecular weight dextran, which was detected in tubular epithelial cells in case of disruption of the glomerular filter (78). By now, several assays to quantify glomerular filtration have been established. Amongst these are *in vivo* fluorescence measurement in the eye or in large vessels in time-laps experiments after injection of fluorescence-labeled 10-, 70-, and/or 500-kDa dextrans (79). Another approach employs transgenic fish expressing eGFP-labeled vitamin D-binding protein (eGFPDBP) of a molecular weight of 78 kDa in the liver, which is repelled from glomerular filtration in healthy fish but leaks into urine in states of glomerular damage and can be quantified in the eye (accumulation in control) and—as excreted protein—in the water surrounding the fish (80).

## Organoids

Generation of kidney organoids by differentiation of pluripotent stem cells (IPS cells) or re-aggregation of single cell suspensions of embryonic kidney cells in culture provided an important new tool for the study of kidney development and disease (81, 82).

However, the delicate morphology of the glomerular filter as well as the need for specific cellular interactions and vascularization has hampered the study of glomerular biology on kidney organoids. In addition, recent single cell transcriptomic analysis of organoids has identified incomplete differentiation of all kidney organoid cell types, including podocyte progenitors (83).

In an elegant study, researchers generated organoids mixing murine embryonic kidney cells and implanted these organoids into nephrectomized athymic rats. They describe differentiation of morphologically and functionally intact glomeruli (84). In addition, the authors were able to integrate human amniotic fluid stem cells into chimeric organoids by mixing murine embryonic kidney cells and human amniotic fluid stem cells before *in vitro* organoid culture. Interestingly, also human amniotic fluid stem cells generated functional podocytes. Similar results were recently confirmed for human pluripotent stem cell derived organoids transplanted under the renal capsule of immunocompromised mice (85).

In conclusion, organoids will become a very important tool also in glomerular research. Again the complexity of glomerular structure and interaction of glomerular cell types is the major challenge to overcome.

## Conclusion

Research of the last two decades has boosted our understanding of podocyte cell biology and genetics and provides growing understanding of the composition of the renal filtration barrier and cellular interactions needed to maintain its function. Experimental work in podocyte cell culture models informed on expression, trafficking, interaction and turnover of essential proteins of podocyte function. However, cell culture studies in podocytes are limited due to the fact that podocytes in culture lose their characteristic features. Cultured podocytes lack the intricate foot process morphology, cell polarity is incompletely preserved, and most importantly, intercellular contacts are neither structurally nor functionally close to the slit-diaphragm found *in vivo*. It is very clear that *in vivo* models are needed to understand glomerular physiology and to address podocyte diseases experimentally. Besides indispensable rodent models, model organisms like *C. elegans, Drosophila melanogaster*, and the zebrafish have entered the stage of glomerular research and allow unparalleled functional analyses of inter-cellular interactions and morphogenesis, signaling mechanisms, cell polarity, and filtration *in vivo*. Kidney organoids may become an additional important tool in the future.

## Author contributions

All authors listed have made a substantial, direct and intellectual contribution to the work, and approved it for publication.

### Conflict of interest statement

The authors declare that the research was conducted in the absence of any commercial or financial relationships that could be construed as a potential conflict of interest.
